# Leisure-time physical activities and the risk of cardiovascular mortality in the Malmö diet and Cancer study

**DOI:** 10.1186/s12889-021-11972-6

**Published:** 2021-10-26

**Authors:** Sara Bergwall, Stefan Acosta, Stina Ramne, Pascal Mutie, Emily Sonestedt

**Affiliations:** 1 Department of Clinical Sciences Malmö, Lund University, Malmö, Sweden; 2 Vascular Centre, Department of Cardio-Thoracic and Vascular Surgery, Skåne University Hospital Malmö, Malmö, Sweden

**Keywords:** Leisure-time physical activity, High-intensity physical activity, Cardiovascular mortality, Running, Prevention

## Abstract

**Background:**

The association between leisure-time physical activity and cardiovascular mortality has been previously studied, but few studies have focused on specific activities and intensities.

**Methods:**

The association between different leisure-time physical activities and cardiovascular mortality was investigated among 25,876 individuals without diabetes or cardiovascular disease from the population-based Malmö Diet and Cancer Study cohort. The individuals estimated the average duration spent on 17 physical activities at baseline in 1991–1996 and after 5 years. Cardiovascular mortality was obtained from a register during a mean of 20 years of follow-up.

**Results:**

A total leisure-time physical activity of 15–25 metabolic equivalent task (MET) hours/week was associated with a decreased risk of cardiovascular mortality (HR 15–25 vs < 7.5 MET-h/week =0.80, 95% CI 0.69–0.93), with no further risk reduction at higher levels. Several high-intensity activities (i.e., lawn tennis and running) and moderate-intensity activities (i.e., golf, cycling and gardening) were associated with a reduced risk. Individuals who engaged in high-intensity physical activity for an average of 2.29 MET h/week (30 min/week) had an 18% (95% CI 0.72–0.93) reduced risk of cardiovascular mortality compared with non-participants, and no further risk reductions were observed at higher levels. Decreased risk was observed among individuals who had started (HR 0.56, 95% CI 0.32–0.97) or continued (HR 0.49, 95% CI 0.36–0.66) high-intensity activities at the five-year follow-up.

**Conclusions:**

Moderate- and high-intensity leisure-time physical activities reduced the risk of cardiovascular mortality. With regard to total leisure-time physical activity, the largest risk reduction was observed for 15–25 MET-h/week (equivalent to walking for approximately 5 h/week).

## Background

In 2011, the World Health Organization (WHO) developed an action plan containing nine targets aimed at combating non-communicable diseases. One of the targets is to reduce the global rate of inadequate physical activity by 10% by 2025 [1]. The WHO recommends that each adult perform at least 150–300 min of moderate-intensity aerobic physical activity or at least 75–150 min of high-intensity physical activity per week, as well as participate in muscle-strengthening activities at least 2 days a week for additional health benefits. It is also recommended that adults limit their time spent being sedentary [2]. Today, however, it is estimated that a quarter of the world’s population is insufficiently physically active, and this number is expected to increase in the coming years, especially in high-income countries [3].

Studies focusing on the prevention of cardiovascular mortality are pertinent because ischaemic heart disease and stroke are the most common causes of death, and in 2016, these diseases accounted for over 15 million deaths worldwide [4]. A systematic review of 44 prospective observational studies, with 19 studies that were eligible for inclusion in a dose-response analysis, found a negative linear correlation between leisure-time physical activity and cardiovascular mortality, and that association that remained after adjusting for several potential confounders [5]. While the association between total leisure-time physical activity and cardiovascular mortality has been studied extensively [5–10], studies on specific physical activities reporting the associations between frequencies or intensities and cardiovascular health are scarce. A recent systematic review concluded that moderate evidence exists regarding the cardiovascular health benefits of running and football, but for other activities, the results were unclear, and many of the included studies were cross-sectional. The review also suggested that it was primarily high-intensity leisure-time physical activity that has beneficial effects on cardiovascular health [11]. However, a later cohort study identified participation in swimming, racquet sports and aerobics as being associated with a reduced risk of cardiovascular mortality, while no associations were found for running, football and cycling. Potential explanations provided by the authors for the non-significant results were the low numbers of mortality events in the exposure group for running and limited number of participants in the exposure group for football [12].

The primary aims of this study were to investigate the associations of cardiovascular mortality with total leisure-time physical activity and 17 different types of physical activity in a large cohort with 20 years of follow-up. As a secondary aim, this study was performed to examine whether the physical activity patterns of the included individuals had changed 5 years after baseline and whether this had impacted their risk of cardiovascular mortality.

## Method

### Study population and data collection

**Fig. 1:**
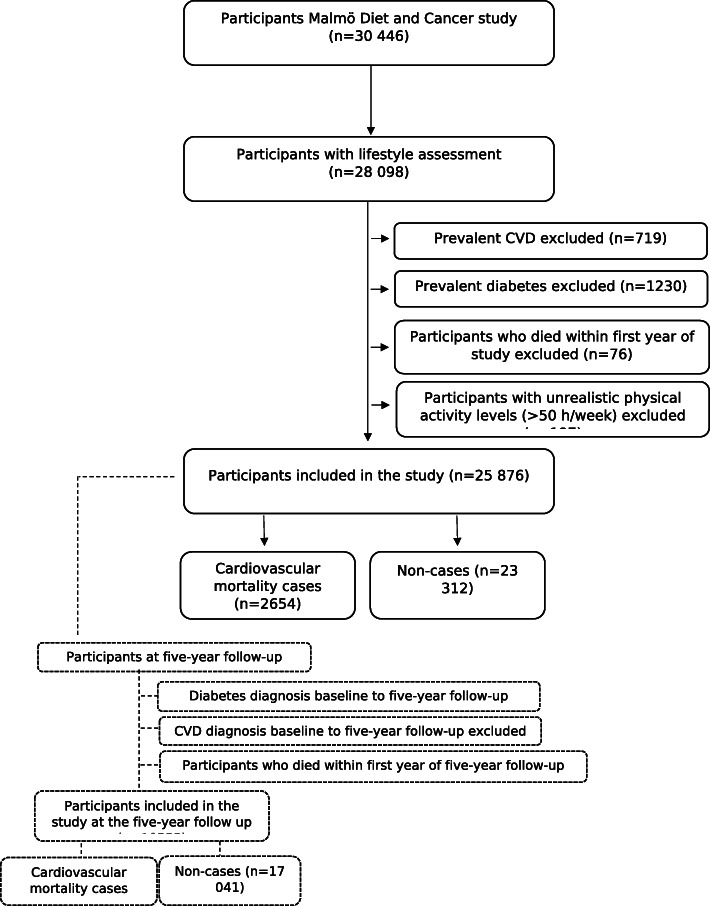
Descriptive flow diagram of study participants and exclusions. Some individuals had multiple exclusion criteria

The Malmö Diet and Cancer Study (MDCS) is a prospective cohort study for which baseline data collection was carried out between 1991 and 1996. In total, the cohort consists of 30,446 men and women born between 1923 and 1950. The recruitment and data collection procedures have previously been described [13, 14]. Among the participants, 28,098 individuals completed an extensive lifestyle questionnaire, underwent anthropometric measurements, and completed dietary assessments. We excluded individuals with prevalent diabetes (*n* = 1230) or cardiovascular disease (*n* = 719) at baseline, individuals who died within the first year of follow-up (*n* = 76) and those with unrealistic leisure-time physical activity levels exceeding > 50 h/week (*n* = 197). Some individuals had multiple exclusion criteria. This resulted in a study population of 25,876 individuals (Fig. 1). All study participants gave written informed consent, and the study was approved by the Regional Ethics Review Board in Lund, Sweden (Dnr §LU5190).

### Physical activity variables

At baseline, the participants were asked to estimate the average number of minutes per week spent on 17 common physical activities in their spare time (including transportation to and from work) during each season of the year (spring, summer, autumn, winter). The results from the questionnaire have been previously compared with the results obtained from an accelerometer for a random sample of 369 participants in the MDCS, and moderate correlations between the two methods were found [15]. A list of the 17 activities as well as the metabolic equivalent task (MET) values, as defined by Ainsworth et al. [16], can be found in Table 1. METs estimate the energy expenditure and amount of oxygen required for specific activities. One MET is equal to the energy expended when sitting quietly [16]. Participants were also given the opportunity to freely report their type of activity if none of the 17 alternatives were correct. This activity was then recoded as one of the 17 listed activities with the same estimated MET value.

**Table 1:** The 17 physical activities and the MET values

Activities	MET-values
**Orienteering**	9.0
**Walking up stairs**	8.0
**High intensity**	7.4
**Lawn tennis**	7.0
**Running**	7.0
**Soccer**	7.0
**Swimming**	7.0
**Ball sports**	5.6
**Ballroom dancing**	5.5
**Grass cutting**	5.5
**Digging**	5.0
**Badminton**	4.5
**Folk dancing**	4.5
**Golf**	4.5
**Cycling**	4.0
**Gardening**	4.0
**Gymnastics**	4.0
**Table tennis**	4.0
**Walking**	3.5

MET-values as defined by Ainsworth et al. [16]. Mean value of included activities for ball sports and high intensity

Total leisure-time physical activity was expressed as MET-h/week by multiplying the sum of the MET values for each of the 17 activities by the time spent on each activity. Total leisure-time physical activity (MET-h/week) was divided into five groups: < 7.5, 7.5–15, 15–25, 25–50 and > 50. The cut-offs were set to reflect high and low physical activity levels while still maintaining an adequate number of individuals in each group. Previous research has shown that the largest risk reduction was seen for individuals with low MET values [17], and therefore, the divisions for total leisure-time physical activity in the present study were made to allow for appropriate analyses of these individuals.

A composite variable of high-intensity leisure-time physical activity was created including the following activities: running, swimming, lawn tennis, soccer, and orienteering. All these activities had an estimated MET factor > 6, which is defined as high-intensity physical activity [16]. Walking up stairs, which can sometimes also be considered a high-intensity leisure-time physical activity, was not included in the composite variable because walking up stairs can vary in both definition and intensity between individuals. For the same reason, walking was also not included in further analyses. The variables for ball sports include badminton, table tennis, soccer, and lawn tennis. The most common activities were also categorised into tertiles based on the MET-h/week score, with the non-participants in a separate category. More than 10% of the study population was required to participate at baseline to allow the relevant statistical analyses.

After approximately 5 years, a questionnaire containing the same physical activity questions as in the baseline questionnaire was sent to all individuals who were still alive and living in Sweden. For the present study, the mean time between baseline and the five-year follow-up was 4.36 years and ranged from three to 8 years. The physical activity level reported at the five-year follow-up was compared with the level from the baseline questionnaire. This comparison was made for high-intensity physical activity as well as for the most common activities. Individuals who had not participated in an activity at baseline or after 5 years were categorised as ‘never’. Individuals who had participated in an activity at baseline but had discontinued that activity after 5 years were classified as ‘stopped’, and individuals who did the opposite, i.e., had not taken part in an activity at baseline but had begun by the five-year follow-up, were classified as ‘started’. Individuals who were participating in an activity at both baseline and the five-year follow-up were categorised as ‘continued’. The date of the five-year follow-up was set as the starting point for the relevant analyses. Information at the five-year follow-up was missing for 6513 individuals. Individuals who received a diagnosis of diabetes (*n* = 458) or cardiovascular disease (*n* = 396) between baseline and the five-year follow-up were excluded from these analyses. Individuals who died within 1 year of the five-year follow-up (*n* = 99) were also excluded from the analyses. Some individuals had multiple exclusion criteria. This resulted in a study population of 18,555 at the five-year follow-up.

### Other variables

To attain information on the participants’ age and sex, individuals’ civic registration numbers were used. The information for all lifestyle variables was gathered from the lifestyle questionnaire completed by the participants at baseline or from the 7-day food diary. For alcohol consumption, the individuals who reported that they had not consumed any alcohol in the last year were categorised as non-consumers. The remaining participants were divided into sex-specific quintiles based on their alcohol consumption reported in the 7-day diary. Smoking status was categorised as never, current, or former smoker. Information on smoking status was missing for seven individuals. Information on highest attained education was categorised as follows: less than 9 years, elementary school (9–10 years), upper secondary school (11–13 years), university without a degree and university degree. Information on education level was missing for 28 individuals.

Total energy intake, expressed as kcal/day, was calculated by combining the reported food and drink intake and alcohol included with a food composition database. A diet index was previously developed [18] and ranges from 0 to 6, where a score of 6 equals the most favourable and healthy diet. The dietary components included in the index were polyunsaturated fat, fish and shellfish, sucrose, dietary fibre, saturated fat, and fruit and vegetables. Nurses performed anthropometric measurements at baseline, which included height, weight, and blood pressure after 10 min of rest. Body mass index (BMI) was calculated using the formula kg/m^2^, and these data were missing for 30 individuals. The definition of hypertension was a systolic blood pressure ≥ 130 mmHg and/or diastolic blood pressure ≥ 85 mmHg or if an individual used anti-hypertensive medication. Information on hypertension was not available for 40 of the study participants. The screening date refers to the year and date that the individual filled in the baseline questionnaires and underwent the anthropometric measurements.

### Endpoint ascertainment

Mortality was obtained by linking the civic registration number of each study participant with the death cause register, where International Classification of Diseases (ICD) codes for the underlying main and contributory causes of death were registered. Codes 390–459 from ICD version 9 and I00 – I99 from ICD version 10 identified cardiovascular causes of death, and only those participants registered with such a code as their main cause of death were recorded as having experienced the endpoint of cardiovascular mortality. The last date of follow-up was December 31st, 2016.

#### Validation of cardiovascular mortality

One hundred participants with the main cause of death registered as cardiovascular death according to the cause of death register were randomly selected for the validation procedure using patient record data. Eighty patient records, including 17 autopsy protocols, were used for validation. Among 100 participants, 88 died from a cardiovascular cause, six did not, and six had unclear causes of death. The cardiovascular causes of death were heart failure (*n* = 26), stroke (*n* = 26), ischaemic heart disease (*n* = 23), ruptured aortic aneurysm (*n* = 5), pulmonary embolism (*n* = 3), hypertension (*n* = 2), ruptured gastroduodenal aneurysm (*n* = 1), aortic dissection, type A (*n* = 1) and aortic valve stenosis (*n* = 1). The non-cardiovascular causes of death were pneumonia (*n* = 3), chronic obstructive pulmonary disease (*n* = 2) and infection of unclear origin (*n* = 1). Hence, cardiovascular mortality was confirmed in 94% (88/94) of the participants.

### Statistical analyses

The baseline characteristics of the participants according to groups of leisure-time physical activity were expressed as the mean (standard deviation) for age, BMI, and diet index and as the count (%) for the remainder of the baseline variables. To attain the pertinent hazard ratios (HRs), Cox proportional hazards regressions were constructed using different models. The basic model included age, sex, and screening date. The multivariable model included age, sex, education, smoking, alcohol, diet index, total energy intake and screening date. These covariates were identified from the literature to be potential confounders. BMI, which might be considered both a confounding factor and a mediating factor, was further added to the multivariable model and tested. In supplementary analyses, hypertension was added to the multivariable model including BMI. Follow-up time was used as the time scale, and cardiovascular mortality was the outcome variable.

The proportional hazard assumption was tested by plotting Schoenfeld’s residuals. The variables that violated the proportional hazard assumption were age, sex, diet index, hypertension, total leisure-time physical activity, orienteering, cycling, and gardening. These variables were stratified and tested in the basic and multivariable models, with unchanged results. All statistical analyses were carried out in SPSS version 25 (SPSS Inc., Chicago, IL, USA) and Rstudio 1.3.959 (RStudio, Boston, MA, USA) using R 4.0.2. Potential collinearity between the 17 activities were tested, tolerance was set at 0.7 and no major collinearity issues were identified. The chosen level of statistical significance was 0.05.

## Results

### Baseline characteristics

Table 2 shows the baseline characteristics for the lowest, middle, and highest groups of leisure-time physical activity. Similar age and sex distributions across categories were observed. The category with the lowest physical activity had 10% who were non-consumers of alcohol, while the category with the highest physical activity had 6%. Hypertension at baseline was prevalent in 76–78% of the participants across the categories.

**Table 2:** Baseline characteristics according to categories of total leisure-time physical activity (expressed in MET-h/week) in the Malmö Diet and Cancer Cohort

	Category 1 (≤7.5 MET-h/week)	Category 2 (7.5–15 MET-h/week)	Category 3 (15–25 MET-h/week)	Category 4 (25–50 MET-h/week)	Category 5 (> 50 MET-h/week)
**n**	2434	3869	5953	9490	4130
**Age at baseline**	57.6 (7.4)	57.4 (7.4)	57.6 (7.5)	57.7 (7.7)	59.0 (7.9)
**Women (%)**	60	62	64	64	58
**Alcohol consumption (%)**					
Non-consumers	10	7	6	5	6
Quintile 1	22	19	17	18	19
Quintile 3	16	19	19	20	19
Quintile 5	17	18	19	20	19
**Current smokers (%)**	29	31	32	34	36
**Education (%)**					
Less than 9 years	53	44	39	38	42
Upper secondary school (11–13 y)	8	9	8	10	9
University degree	10	13	16	16	14
**Diet index**	2.5 (1.3)	2.7 (1.3)	2.8 (1.4)	3.0 (1.4)	3.0 (1.4)
**BMI**	26.3 (4.5)	26.0 (4.1)	25.6 (3.9)	25.4 (3.8)	25.4 (3.6)
**Hypertension (%)**	78	78	76	75	77

Age, BMI and diet index are shown as the mean (SD), and other variables are shown as %

### Association between total leisure-time physical activity and the risk of cardiovascular mortality

There was an inverse association between total leisure-time physical activity and cardiovascular mortality in the basic model and the multivariable model, with HRs per category of 0.93 (95% CI 0.90–0.96) and 0.96 (95% CI 0.96–0.99), respectively (Table 3). The highest versus the lowest category showed associations in both the basic (HR 0.74 95% CI 0.64–0.84) and the multivariable model (HR 0.82 95% CI 0.71–0.96). The largest risk reduction was seen in the 15–25 MET-h/week category (HR 0.80 95% CI 0.69–0.93) in the multivariable model. When BMI was included in the multivariable model, the inverse association was attenuated (HR 0.97 95% CI 0.94–1.00 per category, HR 0.87 95% CI 0.75–1.02 for highest vs. lowest category). Per category, the associations were similar for men (HR = 0.97; 95% CI = 0.92–1.02) and women (HR = 0.98; 95% CI = 0.94–1.02) in the multivariable model. The highest versus lowest category had HRs of 0.78 (95% CI 0.63–1.00) for women and 0.98 (95% CI 0.80–1.22) for men in the multivariable model.

**Table 3:** Categories of total leisure-time physical activity (expressed as MET-h/week) and HR and 95% CI for the risk of cardiovascular mortality

Total leisure-time physical activity	< 7.5 MET-h/week	7.5–15 MET-h/week	15–25 MET-h/week	25–50 MET-h/week	> 50 MET-h/week	Trend (per category)
**N total/n deaths**	2434/264	3869/397	5953/533	9490/905	4130/465	
**Basic model**^**a**^	1.00	0.90 (0.77–1.05)	0.74 (0.64–0.86)	0.74 (0.64–0.84)	0.74 (0.64–0.86)	0.93 (0.90–0.96)
**Multivariable model**^**b**^	1.00	0.94 (0.81–1.10)	0.80 (0.69–0.93)	0.83 (0.73–0.96)	0.82 (0.71–0.96)	0.96 (0.92–0.99)
Women	1.00	0.86 (0.68–1.07)	0.76 (0.62–0.94)	0.86 (0.71–1.05)	0.78 (0.63–1.00)	0.97 (0.92–1.02)
Men	1.00	1.07 (0.86–1.34)	0.93 (0.75–1.15)	0.91 (0.74–1.11)	0.98 (0.80–1.22)	0.98 (0.94–1.02)
**Multivariable model incl. BMI**^**c**^	1.00	0.96 (0.82–1.13)	0.84 (0.72–0.97)	0.87 (0.76–1.01)	0.87 (0.75–1.02)	0.97 (0.94–1.00)

^a^ Adjusted for age, sex and screening date

^b^ Adjusted for age, sex, screening date, education, smoking status, alcohol, diet index and total energy intake

^c^ Adjusted for age, sex, screening date, education, smoking status, alcohol, diet index, total energy intake and BMI

### Association between participating in different activities and the risk of cardiovascular mortality

The baseline characteristics of the individuals participating in each of the 17 activities are presented in Table 4. The most noticeable sex differences were seen for gymnastics (76% women) and soccer (89% men). The age and BMI values were not considerably different across the activities.

**Table 4:** Baseline characteristics divided according to participation in different activities

Activities	N total (%)/n deaths (%)	Women, %	Current smokers, %	University degree, %	BMI, kg/m^**2**^	Age, years
**Orienteering**	110 (0.4)/4(0.2)	45	45	30	25.2 (3.5)	56.7 (6.8)
**Walking up stairs**	13,823(53)/1182(46)	64	33	16	25.4 (3.9)	56.9 (7.4)
**High intensity**	7563(29)/559(22)	59	37	22	25.3 (3.6)	56.5 (7.4)
**Lawn tennis**	666(3)/49(2)	28	38	28	25.3 (3.2)	57.0 (7.5)
**Running**	2621(10)/118(5)	45	42	26	24.5 (3.0)	54.0 (6.3)
**Soccer**	274(1)/17(1)	11	37	12	26.0 (3.3)	54.0 (5.8)
**Swimming**	5151(20)/430(17)	68	35	21	25.6 (3.8)	57.4 (7.6)
**Ball sports**	1646(6)/125(5)	26	39	21	25.6 (3.4)	56.1 (7.2)
**Ballroom dancing**	2644(10)/214(8)	59	32	14	25.3 (3.6)	56.4 (7.4)
**Grass cutting**	7735(30)/804(31)	40	37	15	25.7 (3.6)	58.4 (7.4)
**Digging**	5646(22)/595(23)	46	36	15	25.7 (3.7)	58.4 (7.5)
**Badminton**	603(2)/45(2)	32	39	21	25.4 (3.19)	55.3 (7.0)
**Folk dancing**	1016(4)/85(3)	61	32	7	25.5 (3.5)	58.5 (7.3)
**Golf**	1946(8)/114(4)	55	42	26	24.7 (3.2)	55.8 (7.0)
**Cycling**	15,938(62)/1415(55)	63	35	16	25.4 (3.7)	57.4 (7.5)
**Gardening**	12,063(47)/1145(45)	57	35	16	25.6 (3.7)	58.1 (7.7)
**Gymnastics**	5953(23)/425(17)	76	36	21	24.8 (3.5)	56.6 (7.8)
**Table tennis**	280(1)/22(1)	17	39	16	25.9 (3.9)	56.6 (7.2)
**Walking**	22,088(85)/2168(85)	64	33	15	25.5 (3.9)	57.9 (7.7)

BMI and age are shown as the mean (SD), and other variables are shown as %. for ball sports and high intensity, the mean MET value is shown

When examining the 17 activities separately, individuals participating in lawn tennis, golf, running, gymnastics, cycling, dancing, grass cutting, digging, and gardening had a lower risk of cardiovascular mortality than individuals not participating in those specific activities after adjusting for potential confounders (Table 5). The strongest association was observed for running, with an HR of 0.64 (95% CI 0.53–0.77) in the multivariable model when comparing participants with non-participants. When including BMI in the model, the association was attenuated for gymnastics and remained virtually the same for the other activities. The results were attenuated but remained significant for lawn tennis, golf, running, cycling and grass cutting when the activities were mutually adjusted for each other. Inverse associations could also be observed for individuals participating in ball sports (HR 0.78; 95% CI 0.65–0.93 for participation vs non-participation) and high-intensity leisure-time physical activity (HR 0.82; 95% CI 0.74–0.90 for participation vs. non-participation) in the multivariable model.

**Table 5:** HRs and 95% CIs for participation in different activities and the risk of cardiovascular mortality

Activities	Basic model^**a**^	Multivariable model^**b**^	Multivariable model incl. BMI^**c**^	Mutually adjusted model^**d**^
**Orienteering**	0.36 (0.13–0.95)	0.43 (0.16–1.16)	0.44 (0.17–1.18)	0.51 (0.19–1.37)
**Walking up stairs**	0.92 (0.85–0.99)	0.95 (0.87–1.02)	0.95 (0.88–1.03)	0.98 (0.91–1.07)
**High intensity**	0.75 (0.68–0.83)	0.82 (0.74–0.90)	0.82 (0.75–0.91)	
**Lawn tennis**	0.62 (0.47–0.82)	0.67 (0.50–0.89)	0.67 (0.50–0.90)	0.73 (0.54–0.97)
**Running**	0.56 (0.47–0.68)	0.64 (0.53–0.77)	0.67 (0.55–0.81)	0.71 (0.59–0.86)
**Soccer**	0.93 (0.57–1.50)	0.94 (0.58–1.52)	0.93 (0.58–1.51)	1.03 (0.64–1.68)
**Swimming**	0.86 (0.77–0.95)	0.91 (0.82–1.02)	0.91 (0.82–1.01)	0.97 (0.87–1.08)
**Ball sports**	0.73 (0.61–0.87)	0.78 (0.65–0.93)	0.78 (0.65–0.94)	
**Ballroom dancing**	0.90 (0.78–1.04)	0.90 (0.78–1.03)	0.91 (0.79–1.05)	0.96 (0.84–1.11)
**Grass cutting**	0.75 (0.69–0.82)	0.80 (0.73–0.88)	0.81 (0.74–0.88)	0.87 (0.78–0.96)
**Digging**	0.83 (0.75–0.91)	0.86 (0.78–0.95)	0.87 (0.79–0.95)	0.98 (0.88–1.10)
**Badminton**	0.77 (0.57–1.03)	0.81 (0.60–1.09)	0.82 (0.61–1.11)	0.92 (0.68–1.25)
**Folk dancing**	0.78 (0.63–0.97)	0.76 (0.61–0.95)	0.79 (0.63–0.98)	0.81 (0.65–1.01)
**Golf**	0.63 (0.53–0.77)	0.72 (0.59–0.87)	0.73 (0.60–0.89)	0.74 (0.61–0.90)
**Cycling**	0.78 (0.72–0.84)	0.84 (0.77–0.90)	0.85 (0.79–0.92)	0.90 (0.83–0.98)
**Gardening**	0.76 (0.70–0.82)	0.82 (0.76–0.89)	0.83 (0.77–0.90)	0.91 (0.83–1.00)
**Gymnastics**	0.79 (0.72–0.88)	0.87 (0.79–0.97)	0.91 (0.81–1.01)	0.98 (0.88–1.10)
**Table tennis**	0.75 (0.49–1.14)	0.79 (0.51–1.19)	0.77 (0.51–1.18)	0.83 (0.54–1.26)
**Walking**	0.83 (0.75–0.92)	0.91 (0.82–1.01)	0.93 (0.84–1.04)	0.94 (0.84–1.05)

^a^Adjusted for age, sex and screening date

^b^Adjusted for age, sex, screening date, education, smoking status, alcohol, diet index and total energy intake

^c^Adjusted for age, sex, screening date, education, smoking status, alcohol, diet index, total energy intake and BMI

^d^Adjusted for age, sex, screening date, education, smoking status, alcohol, diet index, total energy intake and mutually adjusted for the 17 activities

Inverse associations with cardiovascular mortality for the highest tertile compared with non-participation were identified in the multivariable model for running (HR 0.68 95% CI 0.51–0.90) and for the composite variable of high-intensity leisure-time physical activity (HR 0.82 95% CI 0.69–0.97), as shown in Table 6. Adding BMI to the multivariable model did not change the results.

**Table 6:** HRs and 95% CIs for the most common activities (≥10% participating) and high-intensity leisure-time physical activity divided into tertiles

	No activity	1	2	3	Trend (per category)
**High-intensity exercise**					
N total/n deaths	18,313/2005	3746/268	2066/144	1751/147	
Mean MET-h/week	0	2.29	6.53	18.25	
Mean hours/week	0	0.33	0.93	2.57	
Basic model^a^	1.00	0.74 (0.65–0.84)	0.75 (0.63–0.89)	0.77 (0.65–0.91)	0.88 (0.84–0.93)
Multivariable model^b^	1.00	0.81 (0.71–0.92)	0.82 (0.69–0.97)	0.82 (0.69–0.97)	0.92 (0.87–0.96)
Multivariable model incl. BMI^c^	1.00	0.82 (0.72–0.93)	0.82 (0.69–0.97)	0.84 (0.70–0.99)	0.92 (0.88–0.97)
**Running**					
N total/n deaths	23,255/2446	1031/36	736/34	854/48	
Mean MET-h/week	0	2.25	5.73	16.05	
Mean hours/week	0	0.32	0.82	2.29	
Basic model	1.00	0.52 (0.37–0.72)	0.56 (0.40–0.78)	0.61 (0.46–0.81)	0.79 (0.73–0.87)
Multivariable model	1.00	0.60 (0.43–0.84)	0.63 (0.45–0.88)	0.68 (0.51–0.90)	0.84 (0.77–0.91)
Multivariable model incl. BMI	1.00	0.63 (0.46–0.88)	0.65 (0.46–0.91)	0.71 (0.53–0.94)	0.85 (0.78–0.93)
**Swimming**					
N total/n deaths	20,725/2134	1860/136	1928/157	1363/137	
Mean MET-h/week	0	1.22	3.68	10.88	
Mean hours/week	0	0.17	0.53	1.55	
Basic model	1.00	0.78 (0.65–0.92)	0.83 (0.70–0.97)	1.00 (0.84–1.19)	0.96 (0.91–1.00)
Multivariable model	1.00	0.84 (0.70–0.999)	0.90 (0.76–1.05)	1.03 (0.86–1.22)	0.98 (0.93–1.03)
Multivariable model incl. BMI	1.00	0.85 (0.71–1.02)	0.89 (0.75–1.04)	1.01 (0.85–1.21)	0.97 (0.93–1.02)
**Cycling**					
N total/n deaths	9938/1149	5292/420	5463/489	5183/506	
Mean MET-h/week	0	2.28	7.50	19.80	
Mean hours/week	0	0.57	1.87	4.95	
Basic model	1.00	0.72 (0.64–0.81)	0.77 (0.69–0.85)	0.84 (0.76–0.94)	0.93 (0.90–0.96)
Multivariable model	1.00	0.77 (0.69–0.87)	0.83 (0.74–0.92)	0.90 (0.81–1.01)	0.96 (0.92–0.99)
Multivariable model incl. BMI	1.00	0.79 (0.71–0.89)	0.84 (0.76–0.94)	0.93 (0.83–1.03)	0.96 (0.93–0.998)
**Gymnastics**					
N total/n deaths	19,923/2139	3203/229	962/62	1788/134	
Mean MET-h/week	0	2.26	3.98	8.54	
Mean hours/week	0	0.57	0.99	2.13	
Basic model	1.00	0.81 (0.70–0.93)	0.70 (0.54–0.90)	0.82 (0.69–0.98)	0.90 (0.86–0.95)
Multivariable model	1.00	0.89 (0.78–1.03)	0.76 (0.59–0.97)	0.90 (0.76–1.08)	0.94 (0.89–0.99)
Multivariable model incl. BMI	1.00	0.93 (0.81–1.07)	0.77 (0.60–1.00)	0.94 (0.79–1.12)	0.96 (0.91–1.01)

^a^Adjusted for age, sex and screening date

^b^Adjusted for age, sex, screening date, education, smoking status, alcohol, diet index and total energy intake

^c^Adjusted for age, sex, screening date, education, smoking status, alcohol, diet index, total energy intake and BMI

### Change in leisure-time physical activities after 5 years and risk of cardiovascular mortality

Analyses were also conducted on the change in physical activity status after 5 years for the most common activities and high-intensity leisure-time physical activity (Table 7). In the group of individuals who continued participation, i.e., they were participating in an activity at both baseline and the five-year follow-up, the lowest risk of cardiovascular mortality was identified among those engaging in high-intensity physical activity (HR 0.49 95% CI 0.36–0.66), cycling (HR 0.78 95% CI 0.71–0.90), and gymnastics (HR 0.77 95% CI 0.64–0.92) when compared with the individuals who never participated in those activities. For swimming and running, the lowest HRs were observed for the individuals who began those activities during the five-year time period, with HRs of 0.76 (95% CI 0.60–0.97) and 0.37 (95% CI 0.17–0.77), respectively.

**Table 7:** HRs and 95% Cis for the risk of cardiovascular mortality in those with changes in LTPA after five years for high-intensity activity and the most common activities

	N total/n deaths	Basic model^**a**^	Multivariable model^**b**^	Multivariable model incl. BMI^**c**^
**High-intensity**				
Never	12,467/1130	1.00	1.00	1.00
Stopped	4417/327	0.90 (0.79–1.01)	0.93 (0.82–1.05)	0.92 (0.82–1.05)
Started	321/13	0.57 (0.33–0.98)	0.56 (0.32–0.97)	0.56 (0.32–0.96)
Continued	1350/44	0.44 (0.33–0.60)	0.49 (0.36–0.66)	0.51 (0.37–0.69)
**Running**				
Never	16,127/1424	1.00	1.00	1.00
Stopped	1136/59	0.76 (0.58–0.99)	0.80 (0.62–1.05)	0.83 (0.64–1.08)
Started	337/7	0.36 (0.17–0.76)	0.37 (0.17–0.77)	0.38 (0.18–0.80)
Continued	955/24	0.41 (0.27–0.61)	0.45 (0.30–0.68)	0.48 (0.32–0.72)
**Swimming**				
Never	13,411/1159	1.00	1.00	1.00
Stopped	2013/156	0.96 (0.81–1.13)	0.99 (0.84–1.17)	0.97 (0.82–1.15)
Started	1275/70	0.74 (0.58–0.94)	0.76 (0.60–0.97)	0.76 (0.59–0.96)
Continued	1856/129	0.85 (0.71–1.02)	0.89 (0.74–1.07)	0.90 (0.74–1.08)
**Cycling**				
Never	5141/516	1.00	1.00	1.00
Stopped	2909/295	0.94 (0.82–1.09)	0.98 (0.85–1.13)	0.98 (0.85–1.14)
Started	1425/91	0.79 (0.63–0.99)	0.80 (0.64–1.00)	0.81 (0.65–1.02)
Continued	9080/612	0.75 (0.67–0.84)	0.78 (0.71–0.90)	0.82 (0.73–0.92)
**Gymnastics**				
Never	12,382/1124	1.00	1.00	1.00
Stopped	2127/137	0.93 (0.78–1.11)	0.98 (0.82–1.17)	1.00 (0.84–1.20)
Started	1513/112	0.96 (0.79–1.16)	1.02 (0.84–1.24)	1.04 (0.86–1.26)
Continued	2533/141	0.71 (0.59–0.85)	0.77 (0.64–0.92)	0.80 (0.67–0.96)

^a^Adjusted for age, sex and screening date

^b^Adjusted for age, sex, screening date, education, smoking status, alcohol, diet index and total energy intake

^c^Adjusted for age, sex, screening date, education, smoking status, alcohol, diet index, total energy intake and BMI

### Additional analyses

For all exposure variables, supplementary analyses were conducted in which hypertension was added to the multivariate model including BMI, and the results remained virtually unchanged (data not shown). Post hoc analyses in which the individuals with prevalent diabetes at baseline (*n* = 1230) were included showed unchanged main results (data not shown). Post hoc analyses were also conducted in which the individuals who died of CVD within the first year of follow-up (*n* = 76) showed unchanged main results (data not shown).

## Discussion

The results of the present study showed an association between leisure-time physical activity and a decreased risk of cardiovascular mortality, a result that is corroborated by previously conducted systematic reviews on the topic [5, 19]. Although an association could be established, the overall effect size was small, and the largest risk reductions were primarily identified for activities categorised as high-intense and, in particular, running.

Running was associated with the largest risk reduction, as runners had a 33% lower risk of cardiovascular mortality than non-runners. In addition, the individuals who were running both at baseline and at the five-year follow-up had a 56% reduced risk of cardiovascular mortality when compared with those who reported that they never ran. This finding is inconsistent with a previous report [12] but concurs with a recent systematic review, which concluded that there is substantial evidence for the positive health impacts of running [20]. This review showed that the largest risk reduction for cardiovascular mortality was found in the lowest quintile of running in comparison with non-runners. The lowest quintile of running was characterised by running 1–2 times weekly and < 51 min per week. A slight U-trend regarding cardiovascular mortality risk and the time spent running was also observed, and the authors concluded that to reach the maximum health benefits from running, it is not necessary to engage in extreme events such as marathons [20]. These findings were corroborated in the present study; when runners were divided into tertiles, the largest risk reduction was found in the first tertile, although no U-trend was observed. In the context of the U-trend [21], it is notable that there are data suggesting an increased incidence of atrial fibrillation [22], a disease associated with both heart failure and cardioembolic stroke, myocardial fibrosis [23] and, infrequently, arrhythmogenic sudden death [24] in those involved in extreme physical activities. The greatest mortality benefit was reported to be achieved for individuals who were running and engaged in some other physical activity [25]. The present study accounted for the potential impact of participating in several activities by mutually adjusting for and testing the 17 activities in the models (Table 4). Associations remained significant for running, cycling and grass cutting.

High-intensity physical activity was also associated with a reduced risk of cardiovascular mortality, and individuals in the first tertile had a marginally lower risk than those in tertiles two and three. The individuals in the first tertile were exercising at a high intensity for < 30 min/week. This suggests that the health benefits of high-intensity physical activity do not increase with frequency or duration. Engaging in any high-intensity physical activity is the most important factor and not the duration. It appears that the individuals who reported that they had stopped at the five-year follow-up still had a lower risk than those who had never participated in physical activities. However, the lowest risk was observed for those who were categorised in the ‘continued’ group at the five-year follow-up. Even though associations were found for numerous activities, it was primarily the activities that can be categorised as high-intensity that were associated with the largest risk reduction, which has been corroborated elsewhere [11].

There are several mechanisms and pathways through which physical activity improves the status of many cardiovascular risk factors. Higher levels of physical activity have been shown to, among other benefits, reduce LDL cholesterol [26], blood pressure [27] and C-reactive protein levels [28, 29], all of which are significant intermediators in the development of cardiovascular diseases [29–31]. Physical activity also reduces body weight and promotes a healthy weight distribution, which is especially pertinent, as obesity is a major cause of cardiovascular diseases [32, 33]. Finally, physical activity improves cardiorespiratory fitness, which is also associated with a lower risk of developing cardiovascular disease [34].

There are numerous strengths of the present study. The mean follow-up duration in the cohort was over 20 years, which allowed evaluation of the relationship between exposure and outcome. The exclusion of participants with previous cardiovascular disease at baseline ensured that physical activity was studied in the context of primary prevention. The five-year follow-up enabled a more detailed evaluation of the study participants’ physical activity habits. Additionally, the baseline questionnaire included the most common activities at the time. This made it possible to assess the risk of cardiovascular mortality in those participating in 17 different physical activities, instead of only relying on the overarching and less precise variable of total leisure-time physical activity. Another strength is that registers were used to attain the endpoint data, which ensured nearly complete follow-up of the participants. A further strength is that two sensitivity analyses were conducted in which hypertension and diabetes were accounted for, which yielded unaltered results. Sensitivity analyses in which lipid-lowering medications were not considered were required due to the low number of individuals (< 5%) using these medications in the cohort. Finally, the validity of the endpoint of cardiovascular mortality retrieved from the cause of death register was high (94%) after scrutinizing patient records and autopsy protocols.

This study also has some limitations that need to be addressed. First, the self-reported nature of the data could affect the reliability of the results. However, the previously conducted comparison between the results of the questionnaire and data from accelerometers on a random sample from the MDCS (*n* = 369) showed moderate correlations between the two methods [15]. Suspected over-reporters (> 50 h of physical activity/week) were also excluded from the present study to ensure that these factors did not affect the results. Moreover, as this study explicitly focused on physical activity conducted during leisure time, physical activity at other times, such as occupational physical activity, was not included in any analyses. It has been suggested that the most important aspect of physical activity to consider is that performed during leisure time, as this is when the largest health benefit can be observed [35]. Some of the groups in the analyses at the five-year follow-up were very small, as were the number of individuals engaging in some of the activities (i.e., orienteering), which might affect the reliability of these results. Finally, the present study utilised METs as a measure of physical activity intensity. METs are not individualised but assume a standard energy expenditure and oxygen uptake that might not be accurate for some individuals, which can generate misleading results [16].

It can also be argued that the endpoint of cardiovascular mortality is broad and encompasses many diagnoses, making the results less precise. In this study, information on the specific cardiovascular diagnoses for the entire study cohort was not available, the results could therefore not be stratified, and the role of various cardiovascular endpoints could not be tested. However, the aim was to elucidate the role of leisure-time physical activity in the prevention of cardiovascular mortality, and studies focusing on specific cardiovascular diagnoses with high mortality, such as pulmonary embolism [36], can be conducted in the future to further enhance the knowledge of the potential preventative role of physical activity. The validation procedure of the random sample of this paper showed that only 3.4% had fatal pulmonary embolism, 56.8% had cardiac causes of death, and 29.5% had cerebral causes of death, which means that the results of physical activity relate mainly to cardio-cerebral deaths in the present study. Moreover, future studies should consider various physical activities in addition to the overall measure of physical activity, as the results can vary greatly between activities, as has been shown in the present study.

## Conclusions

This prospective cohort study shows that an inverse association between leisure-time physical activity and cardiovascular mortality exists, and the largest risk reduction was observed for individuals in the 15–25 MET-h/week category. While several high-intensity physical activities were associated with a reduced risk of cardiovascular mortality, running appeared to be associated with the largest risk reduction in the present study.

## Data Availability

The database used in the current study is closed, but researchers with an ethical approval from the Swedish Ethical Review Authority may contact the first author SB to gain access. The questionnaire can be found on the following website: https://www.malmo-kohorter.lu.se/mkc/datainsamling-mkc.

## Abbreviations

BMI: Body Mass Index
CI: Confidence Interval MET
HR: Hazard Ratio
ICD: International Classification of Disease
MET: Metabolic Equivalent Task
MDCS: Malmö Diet and Cancer Study
WHO: World Health Organization
